# Relationship between cerebrospinal fluid protein level and electrophysiologic abnormalities in the acute inflammatory demyelinating polyradiculoneuropathy variant of Guillain-Barré syndrome

**DOI:** 10.3205/000299

**Published:** 2021-09-01

**Authors:** Wajid Jawaid, Rabia Sana, Sumera Rafat Umer, Qamar Nisa, Mehwish Butt, Naila Shahbaz

**Affiliations:** 1 Department of Neurology, Dr. Ruth K. M. Pfau Civil Hospital Karachi & Dow University of Health Sciences, Karachi, Pakistan; 2 Department of Medicine & Allied, Jinnah Medical College Hospital Korangi, Karachi, Pakistan

**Keywords:** Guillain-Barre syndrome, acute inflammatory demyelinating polyradiculoneuropathy, blood-nerve barrier, blood-CSF barrier, CSF protein

## Abstract

**Objective:** Guillain-Barré syndrome (GBS) is an autoimmune disease characterized by weakness in limbs or cranial nerve innervated muscles. Acute inflammatory demyelinating polyradiculoneuropathy (AIDP) is the most common variant. Electrophysiologic abnormalities and elevated cerebrospinal fluid (CSF) protein are frequently present in AIDP, but the relationship between these two parameters is not well known. We aimed to fill this gap by studying this relationship.

**Methods:** This was a prospective cross-sectional study conducted for two years in the Department of Neurology, Dr. Ruth K. M. Pfau Civil Hospital, Karachi, Pakistan. All 90 adult patients with the AIDP variant of GBS were selected. Nerve conduction studies were performed to determine the degree of demyelination through the four electrophysiologic demyelination criteria. The CSF sample was sent to lab immediately after lumbar puncture. SPSS version 20.0 was used. The CSF protein level was measured with mean ±SD. Demyelination criteria were measured in frequency and percentages. Chi-square test was applied to a number of demyelination criteria and T-test/ANOVA was applied on mean CSF protein level.

**Results:** We found a mean CSF protein of 37.41 mg/dl (±3.69) with one demyelination criterion, 81.87 mg/dl (±17.39) with two demyelination criteria, 119.75 mg/dl (±31.42) with three demyelination criteria, and 134.00 mg/dl (±42.87) with four demyelination criteria (P-value <0.001).

**Conclusion:** This study demonstrates a significant relationship between CSF protein levels and degree of demyelination in the AIDP variant of GBS. This is an under-researched area in GBS and this study adds favorably to limited data in this regard.

## Introduction

Guillain-Barré syndrome (GBS) is an autoimmune disease which is characterized by weakness in limbs or cranial nerve innervated muscles. It has an acute or subacute onset and usually presents with decreased or absent deep tendon reflexes. Cerebrospinal fluid and electrophysiologic studies demonstrate characteristic abnormalities in this disorder [1]. The incidence of this disease is approximately 1–2 cases per 100,000 population [2]. There are four common variants of GBS, differentiated by electrophysiologic abnormalities, with different pathological bases: acute inflammatory demyelinating polyradiculoneuropathy (AIDP), acute motor axonal neuropathy (AMAN), acute motor and sensory axonal neuropathy (AMSAN), and Miller Fisher syndrome. AIDP is most common among these variants. Around 90% of all GBS cases in North America and Europe present with AIDP; 53 to 70% of all GBS cases in Asia, Central and South America have the AIDP variant [1]. In Pakistan, studies have reported variable results in terms of frequency of GBS variants. One study found similar results to other regional studies, with 60% of cases having the AIDP variant [3]; a couple of other local studies found AIDP in lesser numbers (46% and 47.3% respectively), although AIDP was still the most common GBS variant in their patients [4], [5].

Damage to the peripheral nervous system and the barriers, including the blood-nerve barrier (BNB) and the blood-cerebrospinal fluid barrier (B-CSF-B), is implicated in GBS. BNB and B-CSF-B are barriers between blood and nerve/CSF that result in a stable environment in their surrounding tissues. CSF proteins are elevated in GBS, presumably due to damage to these barriers [6]. Prolonged distal latencies, slowed conduction velocities, abnormal F-waves and H-reflex, and temporal dispersion and/or conduction block are the well-described abnormalities on electrophysiological studies in AIDP. These were described as diagnostic criteria by Albers and Kelly to determine the degree of demyelination in GBS [7]. Another well demonstrated electrophysiological characteristic of GBS is sparing of sural nerve; this helps in differentiating GBS from other neuropathies [8].

Kanda et al. demonstrated the dysfunction of BNB and B-CSF-B in patients of GBS [9]. Gonzalez-Quevedo et al. proposed that rising CSF protein level can be used as a surrogate measure of dysfunction of these barriers [10]. The purpose of this study is to determine the relationship between electrophysiologic abnormalities in AIDP and CSF protein level. DiCapua et al. conducted a similar study in the USA in 2015 that showed increasing CSF protein level with increasing demyelination [11]. More studies are required in different settings to further establish a possible link between these variables. We aimed to fill this gap by conducting this study in all GBS patients with AIDP who presented to our neurology department in a two-year period from 1^st^ July 2018 to 30^th^ June 2020.

## Methods

This was a prospective cross-sectional study conducted in the Department of Neurology, Dr. Ruth K. M. Pfau Civil Hospital, Karachi, Pakistan, after approval from the institutional review board. All the patients presenting with hyporeflexic or areflexic limbs weakness with or without cranial nerves involvement who were subsequently confirmed to have an AIDP variant of GBS were selected with non-probability consecutive sampling over a period of two years (1^st^ July 2018 to 30^th^ June 2020).

Patients aged more than 18 years were enrolled in the study after taking written informed consent. A descriptive profile of patients was noted on pro forma. 4 ml of CSF sample was collected through lumbar puncture and sent to the lab immediately for detailed report. The CSF protein level was measured by a qualified pathologist using a colorimetric method with pyrogallol red and sodium molybdate as standard reagents [12]. The normal CSF protein range of the lab was 20 to 40 mg/dl.

Nerve conduction studies were performed in the electrophysiology section of the Department of Neurology, Dr. Ruth K. M. Pfau Civil Hospital Karachi, using a Nihon Kohden S2 four channel machine to determine the degree of demyelination. At least 4 motor nerves (median nerve, ulnar nerve, tibial nerve and peroneal nerve), and 3 sensory nerves (median nerve, ulnar nerve and sural nerve) were studied. It was ensured that CSF analysis and nerve conduction studies were performed within 24 hours of each other so that a more accurate relationship could be demonstrated. CSF protein level and demyelination criteria were charted on pro forma along with age, gender, and duration from the onset of the disease.

The inclusion criteria of this study were i) patients with hyporeflexic or areflexic limbs weakness with or without cranial nerves involvement who met the diagnostic criteria for AIDP, ii) age more than 18 years, and iii) nerve conduction study and lumbar puncture performed within 24 hours of each other. On the other hand, i) patients who were already diagnosed with peripheral neuropathy due to any other cause, and ii) those with CSF white blood cell count of greater than 50/µl were excluded from this study.

The demyelination criteria proposed by Albers and Kelly were used in this study [7]:

1. Prolonged distal latency in two or more motor nerves: Distal latency >130% of upper limit of normal.
2. Conduction velocity slowing in two or more motor nerves: Conduction velocity <75% of lower limit of normal.
3. Prolonged late responses: F response and H reflex, in one or more motor nerves: >130% of upper limit of normal.
4. Conduction block/temporal dispersion in one or more motor nerves: a. Conduction block: proximal/distal CMAP area ratio <0.50 b. Temporal dispersion: proximal/distal CMAP duration ratio >1.15.

### Statistical analysis

The collected data were analyzed through SPSS version 20.0. The quantitative variables like age, CSF protein level and duration of symptoms were measured with mean ±SD. The qualitative variables like gender and number of demyelination criteria were measured in frequency and percentages. Effect modifiers like age, gender, and duration of symptoms were stratified to see the effect of these on number of demyelination criteria and mean CSF protein level. Post-stratification chi-square test was applied to the number of demyelination criteria and T-test/ANOVA was applied on mean CSF protein level. P-value <0.05 was taken as significant.

## Results

**Figure 1:**
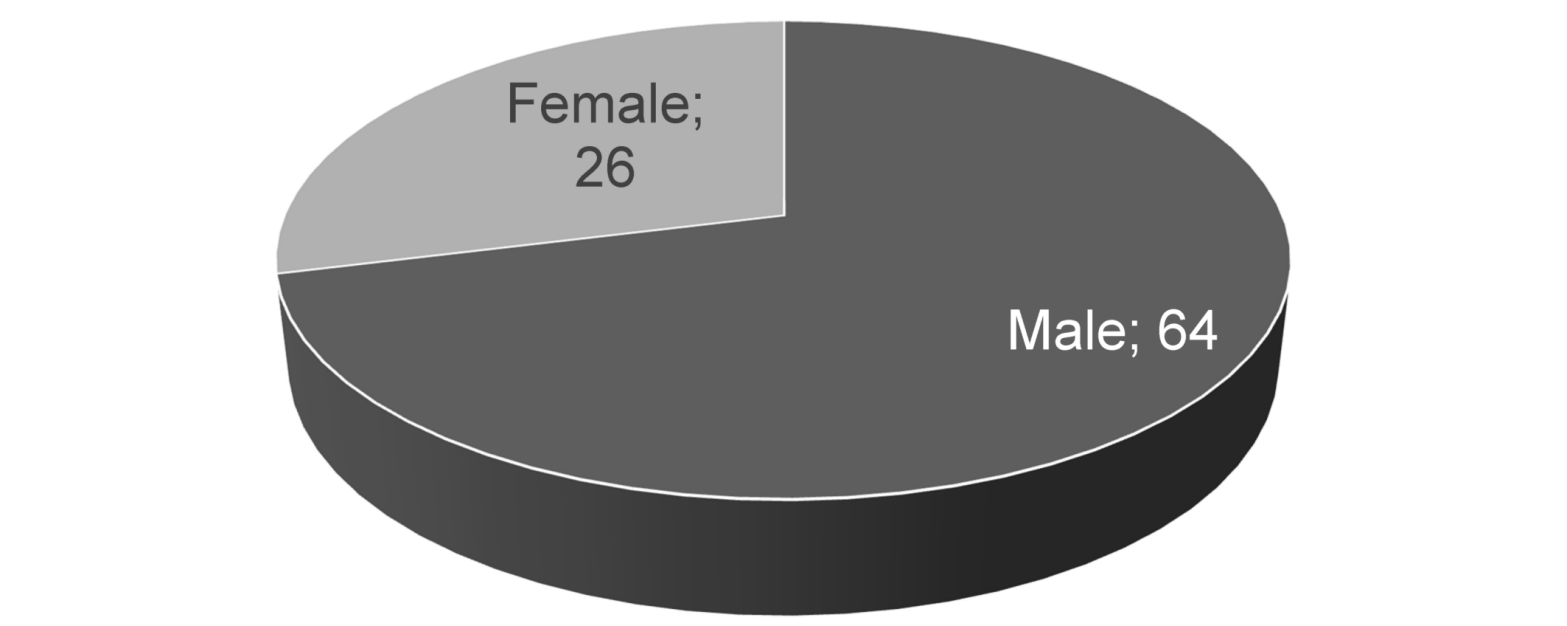
Gender distribution in the study population

**Figure 2:**
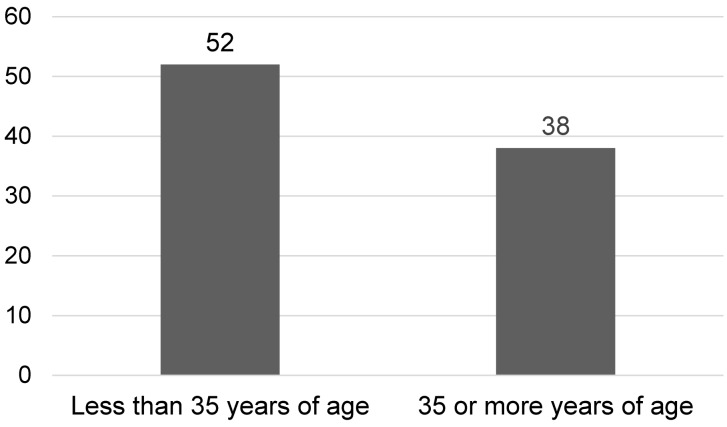
Age distribution in the study population

A total of 90 patients were enrolled in the study. There were 71.1% male and 28.9% female patients (Figure 1 (Fig. 1)). The mean age of patients was 35.1±12.9 years. 57.8% were below 35 years of age and 42.2% were 35 or more years of age (Figure 2 (Fig. 2)). 48.9% of patients presented with less than 10 days history of symptoms and 51.1% had symptoms lasting for 10 or more days at presentation.

**Figure 3:**
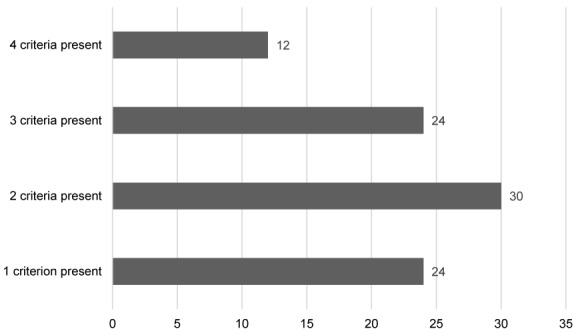
Number of electrophysiologic demyelination criteria in the study population

**Figure 4:**
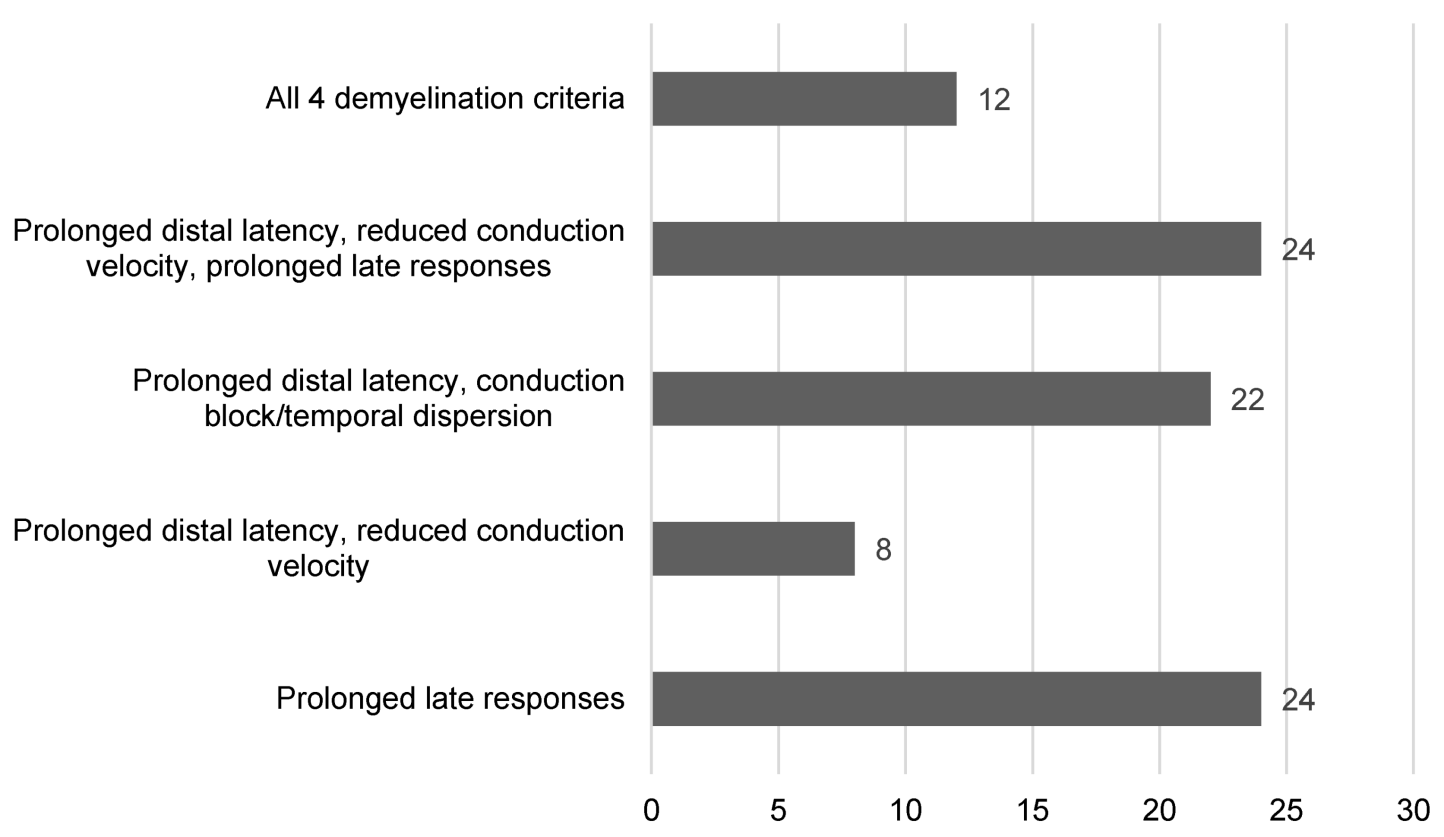
Frequency of specific demyelination findings in the study population

26.6% of patients had one demyelination criterion, 33.3% of patients had two demyelination criteria, 26.6% of patients had three demyelination criteria and 13.3% had four demyelination criteria in nerve conduction studies (Figure 3 (Fig. 3)). Sural nerve sparing pattern was found in 66 (73.3%) patients. The frequency of different demyelination criteria in our patients is shown in Figure 4 (Fig. 4).

We found a mean CSF protein of 37.41 mg/dl (±3.69) with one demyelination criterion, a mean CSF protein of 81.87 mg/dl (±17.39) with two demyelination criteria, a mean CSF protein of 119.75 mg/dl (±31.42) with three demyelination criteria, and a mean CSF protein of 134.00 mg/dl (±42.87) with four demyelination criteria (P-value <0.001). The stratified data of relationship between mean CSF protein and demyelination according to age, gender and duration of disease are shown in Table 1 (Tab. 1), Table 2 (Tab. 2) and Table 3 (Tab. 3).

## Discussion

We found a statistically significant relationship between CSF protein and electrophysiologic abnormalities in the AIDP variant of GBS. In 2015, DiCupua et al. published a retrospective study on the positive association between CSF protein and electrophysiologic findings of demyelination among GBS patients [11]. Bourque et al. in 2020 conducted a study on CSF total protein level among patients with multiple variants of GBS. Their study subjects comprised 134 patients with sensorimotor GBS, 13 patients with a pure motor form of GBS, 8 patients with a localized form of GBS and 18 patients with a Miller Fisher variant of GBS. Their study results concluded that the mean total CSF protein was higher for patients with sensorimotor GBS, localized GBS and acute onset chronic inflammatory demyelinating polyradiculoneuropathy. They also found that the mean CSF protein was significantly more in the demyelinating variant compared to axonal variants [13].

The male and female ratio in our study was 2.4:1. Hughes et al. studied 35 different case series of GBS patients and found a male and female ratio of 1.25:1 [14]. This male preponderance is unusual for an autoimmune disease. Kiliç et al. also had more males in their pediatric GBS study than females, with a ratio of 1.25:1. They also concluded that GBS was most common in spring, followed by summer season [15].

In our study, 26.6% of patients had only one demyelination criterion of prolonged late responses in one or more motor nerves. There were two different pairs of two demyelination criteria: 24.4% of patients had prolonged distal latency in two or more motor nerves and conduction block/temporal dispersion in one or more motor nerves; 8.9% of patients had prolonged distal latency and reduced conduction velocity in two or more motor nerves. 26.6% of patients were found to have three demyelination criteria: prolonged distal latency in two or more motor nerves, reduced conduction velocity in two or more motor nerves, and prolonged late responses in one or more motor nerves. The remaining 13.3% had all four demyelination criteria present in nerve conduction studies.

Either alone or in combination, the most common demyelination criterion in our study was prolonged distal latency in two or more motor nerves which was found in 66 patients. This was followed by prolonged late responses in one or more motor nerves in 60 patients, reduced conduction velocity in two or more motor nerves in 44 patients, and conduction block/temporal dispersion in one or more motor nerves in 34 patients. Das et al. found that all their AIDP patients had prolonged distal latency, and 40% of patients had prolonged F wave latency, which is one of the late responses [16]. Wali et al. studied early electrophysiological findings in the AIDP variant of GBS and found that a vast majority of patients had prolonged late responses in one or more motor nerves [17]. Jin et al. published a study on the early electrophysiologic findings among GBS patients in 2018. Their study results concluded that motor nerves were involved earlier in the course of disease compared to sensory nerves [18].

Sural nerve sparing is considered a fairly specific measure to differentiate GBS from closely related diseases with a specificity of up to 0.96 in GBS patients [7]. Our study supports this observation by finding this pattern in 73.3% of the patients. This finding is agreeable with the viewpoint that there may be merit in adding sural nerve sparing pattern as an electrodiagnostic criterion for GBS [19].

The findings of this study suggest that the CSF protein level correlates with the degree of demyelination in patients with the AIDP variant of GBS. The patients with three demyelination criteria, for example, were found to have more CSF protein on average compared to those with two demyelination criteria. This relationship remained intact with stratifications for age, gender and duration of symptoms.

The limitation of this study is that it included patients from a single center and therefore generalization over the whole population may not be appropriate. The major strength of this study is a significant sample size spread over two years that minimizes the chances of false representation of the actual state of affairs.

Earlier studies have suggested that CSF protein concentration can be used as a surrogate measure of the integrity of the BNB and B-CSF-B. The disruption to these barriers is understood to cause inflammation of the nerve roots, resulting in elevation of the CSF protein level [20]. Consequently, an increase in CSF protein is thought to represent increasing dysfunction of these barriers [6], [9], [10], [11]. Therefore, the relationship between the degree of demyelination and the CSF protein level found in this study points towards the involvement of these barriers in the disease. Currently, immunomodulatory regimens are the mainstay of treatment for GBS. Effective disease-modifying therapies in GBS are currently lacking and there is a dire need to develop these potentially better treatment modalities [21]. If subsequent studies can implicate these barriers in the pathogenesis of GBS, they may prove to be an additional therapeutic target to alter the course of this often debilitating disease.

## Conclusions

This study demonstrates a significant relationship between CSF protein levels and degree of demyelination in the AIDP variant of GBS. This is an under-researched area in GBS and this study adds favorably to limited data in this regard. An increase in CSF protein is considered a surrogate measure of progressive dysfunction of barriers between blood and nerve/CSF. This study points towards these barriers as being key factors in the pathophysiology of this disease by finding that the CSF protein level rises with an increasing degree of demyelination. This may pave the way for targeting these barriers therapeutically in the future if further studies indicate this phenomenon, and may effect a major shift from the immunomodulatory therapy currently offered in this disease with mixed results [22], [23], [24].

## Notes

### Competing interests

The authors declare that they have no competing interests.

## Figures and Tables

**Table 1:**
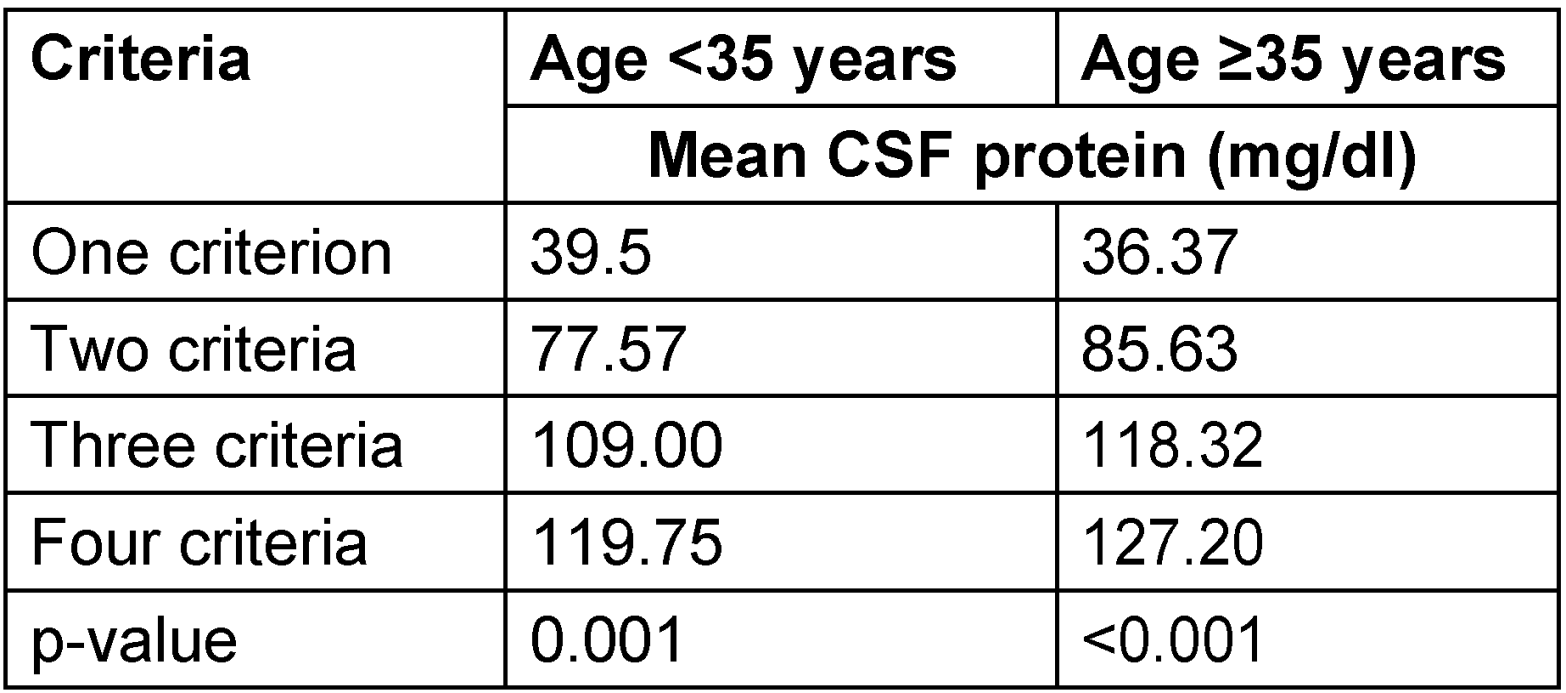
Stratified analysis of relationship between cerebrospinal fluid protein level and electrophysiologic abnormalities in the AIDP variant of Guillain-Barré syndrome by age

**Table 2:**
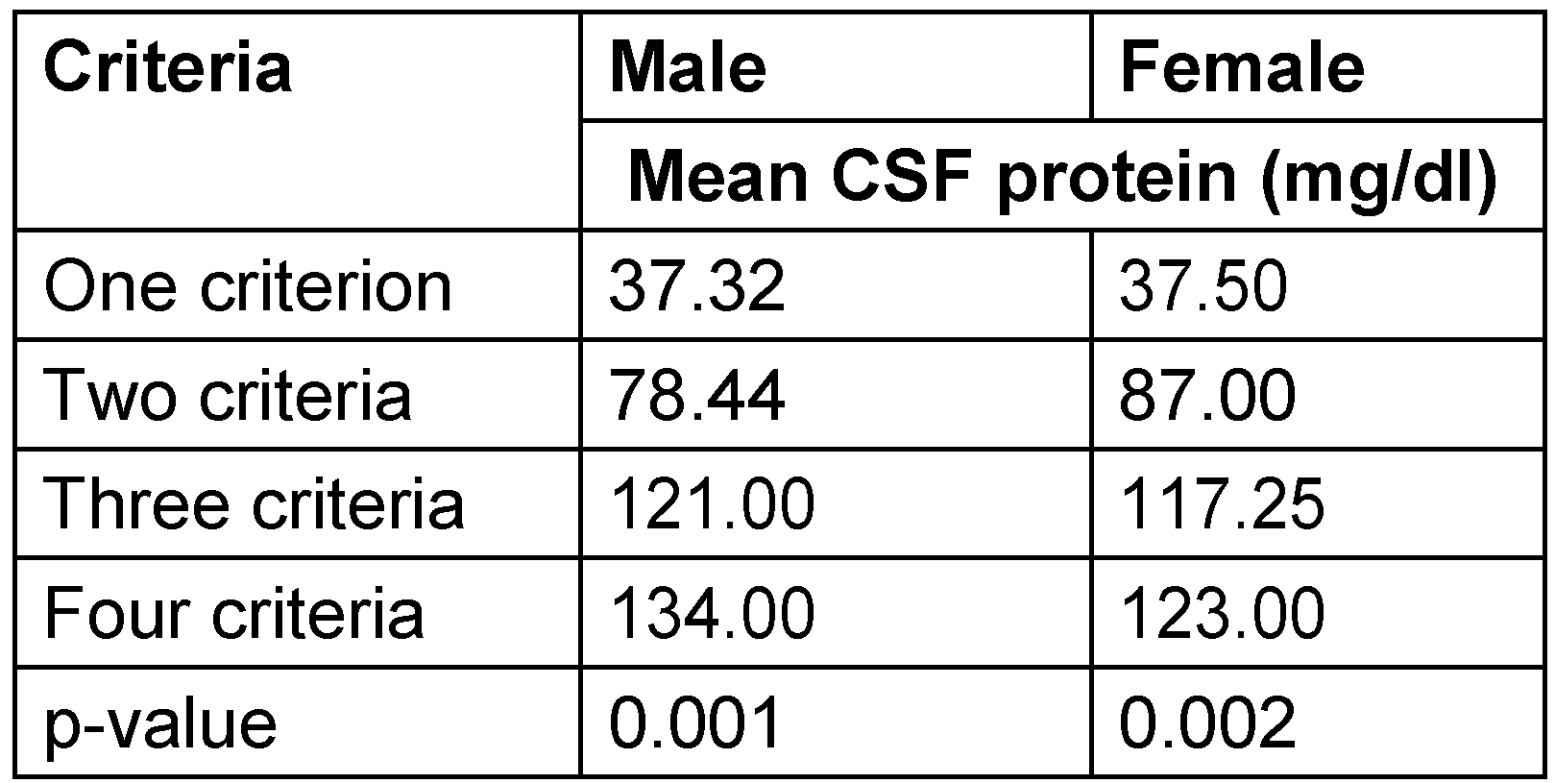
Stratified analysis of relationship between cerebrospinal fluid protein level and electrophysiologic abnormalities in the AIDP variant of Guillain-Barré syndrome by gender

**Table 3:**
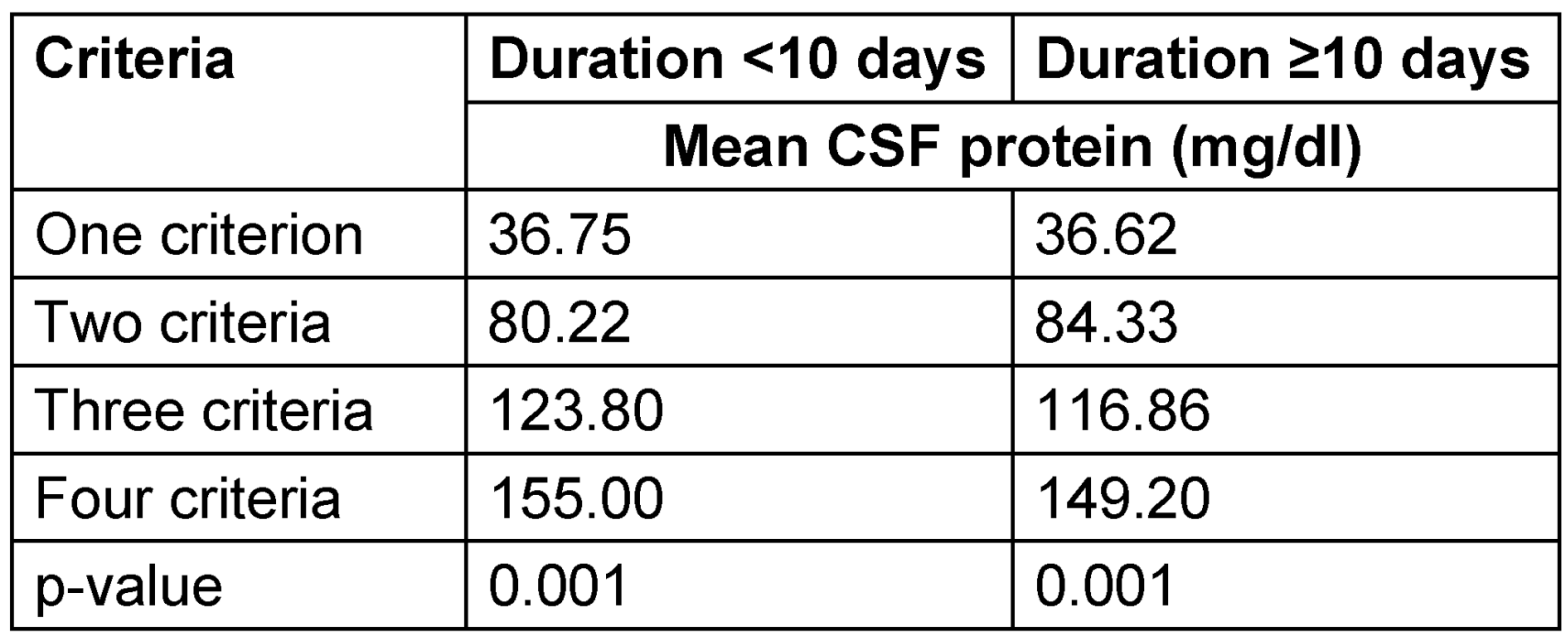
Stratified analysis of relationship between cerebrospinal fluid protein level and electrophysiologic abnormalities in the AIDP variant of Guillain-Barré syndrome by duration of disease
